# Becoming a Mother During COVID-19 Pandemic: How to Protect Maternal Mental Health Against Stress Factors

**DOI:** 10.3389/fpsyt.2021.764207

**Published:** 2022-03-15

**Authors:** Hugo Bottemanne, Brune Vahdat, Cleo Jouault, Ruben Tibi, Lucie Joly

**Affiliations:** ^1^Department of Psychiatry, Sorbonne University, Pitié-Salpêtrière Hospital, DMU Neurosciences, Assistance Publique-Hôpitaux de Paris (AP-HP), Paris, France; ^2^Paris Brain Institute - Institut du Cerveau (ICM), UMR 7225/UMRS 1127, Pitié-Salpêtrière Hospital, Sorbonne University/CNRS/INSERM, Paris, France; ^3^Sorbonne University, Department of Philosophy, SND Research Unit, UMR 8011, Paris, France; ^4^Perinatal Psychiatry Unit, Department of Psychiatry, Robert Ballanger Hospital, GHT Grand Paris Nord-Est, Paris, France

**Keywords:** perinatality, perinatal psychiatry, COVID-19, SARS-CoV-2, mental health, maternal health, neonatal health, postpartum depression

## Abstract

During the COVID-19 pandemic, there were an increasing prevalence of perinatal psychiatric symptoms, such as perinatal anxiety, depression, and post-traumatic stress disorders. This growth could be caused by a range of direct and indirect stress factors related to the virus and changes in health, social and economic organization. In this review, we explore the impact of COVID-19 pandemic on perinatal mental health, and propose a range of hypothesis about their etiological mechanisms. We suggest first that the fear of being infected or infected others (intrauterine transmission, passage of the virus from mother to baby during childbirth, infection through breast milk), and the uncertainty about the effect of the virus on the fetuses and infants may have played a key-role to weakening the mental health of mothers. We also highlight that public health policies such as lockdown, limiting prenatal visits, social distancing measures, and their many associated socio-economic consequences (unemployment, loss of income, and domestic violence) may have been an additional challenge for perinatal mental health. Ground on these hypotheses, we finally purpose some recommendations to protect perinatal mental health during a pandemic, including a range of specific support based on digital technologies (video consultations, phone applications) during pregnancy and the postpartum period.

## Introduction

The COVID-19 pandemic has caused an unprecedented health, social, and economic crisis across the world. Although most biomedical research initially focused on the epidemiology of the disease, the respiratory symptoms caused by Sars-Cov-2 in the adult population, or even potential therapies, new interest has gradually focused on the collateral effects of the pandemic on mental health (1–3). Initial studies have shown an increasing prevalence of mental disorders in the general population during the pandemic (4). Among them, pregnant women and new mothers constitute a specific, vulnerable population, affected in the foreground by the dramatic consequences of the pandemic (5).

Pregnancy and the postpartum period involve profound physiological (somatic and hormonal), psychological (process of motherhood) and social changes in future mothers. Apart from the disorders caused by infection, the impact of policies to fight the pandemic could have affected maternal health, modifying the organization of perinatal care, intra-family relations, or even living conditions (5). Previous studies have shown that patients in the prenatal and postnatal periods are particularly at risk of developing mental disorders during health or social disasters (6). These mental disorders represent major challenges for public health because of their negative impact on the mother and on the subsequent growth and development of the child (1). For example, depression and perinatal anxiety are associated with risks of miscarriage, pre-eclampsia, gestational hypertension, premature birth, lower Apgar scores, low birth weight, postnatal depression, higher and early attachment disorders (7, 8). Knowing the risk of these prenatal and postnatal mental disorders on mothers, fetuses and infants, it is imperative to offer responses to the mental health needs of this population (9).

Maternal fear about the effects of the virus on pregnancy and the fetus adds to the general psychological difficulties encountered during pregnancy, especially fear of birth (10). A large number of mothers, mostly first-time mothers, are terrified by the physiological stages of pregnancy and the prospect of childbirth, dreading each obstretrical follow-up consultation (11). In these mothers, prenatal stress constitutes a major risk factor for postpartum depression and perinatal mental disorders in general (12). For this at-risk population, the consequences of COVID on maternal mental health are even greater.

In this review, we offer an update comparing our clinical experience with the current literature on perinatal mental health during the COVID-19 pandemic. We suggest that a better understanding of the effect of the pandemic on the mental health of pregnant and postpartum women will allow the implementation of early and adapted interventions to deal with it, in order to protect maternal mental health from the stress induced by the pandemic. We offer a series of advice that can be delivered to patients during the perinatal period in order to reduce the stress associated with the pandemic, and improve the coping skills for these mothers. Finally, we suggest several public health measures that can be applied to these specific clinical situations, in order to improve their management.

## Increased Prevalence of Psychiatric Disorders

### Perinatal and Postpartum Anxiety

The perinatal period, including pregnancy through childbirth and the first year postpartum, is a time of high vulnerability for mental health. This rise is particularly crucial for prenatal anxiety, defined as the presence of anxiety symptoms during pregnancy, and postpartum anxiety, defined as the presence of anxiety symptoms within 1 year of childbirth.

These perinatal anxiety symptoms lead to a deterioration in quality of life, insomnia, and can cause subsequent depressive disorders, including postpartum depression. Before the pandemic, previous studies suggested a prevalence of prenatal and postnatal anxiety around 15.2% (13), and 45.7% for associated insomnia (14). This risk was even higher in women who experiences a high medical risk pregnancy, or lives in disadvantaged socio-economic conditions.

Thus, the prevalence of perinatal anxiety has significantly rised during the pandemic. A systematic review including 11,187 participants in China evaluated the impact of COVID-19 on anxiety and depression amoung pregnant women. The results showed that the prevalence of anxiety was 34% (95% CI: 0.26–0.43), and prevalence of both anxiety and depression was 18% (95% CI: 0.09–0.29) (15).

In a meta-analysis reviewing the effect of COVID-19 on maternal mental health the overall pooled State-Trait Anxiety Inventory (STAI) score was significantly higher during pandemic (16). Besides, the COVID-19 pandemic is a unique stressor, with potentially wide-ranging consequences for pregnancy and beyond. The stress induced by the pandemic may have been a major factor in the rise in anxiety symptoms in the perinatal population.

Others studies using different tools to evaluate anxiety showed similar results. Another has compared the mean total Inventory of Depression and Anxiety Symptoms (IDAS II) and Beck Anxiety Inventory (BAI) scores for patients included before COVID-19 and re-evaluated during the outbreak. Interestingly, the mean total IDAS II score have significantly increase during the SARS-CoV-2 pandemic, but with a specific pattern concerning the intensity of the symptoms: the number of patients without anxiety, or with mild anxiety (according to the BAI score), decreased, whereas patients with moderate and severe anxiety increased (17). Few hypotheses have been proposed to explain these specifics of the evolution of anxiety symptoms during the pandemic, and these results need to be clarified.

Studies also report a time effect regarding these symptoms. In a meta-analysis of the worldwide prevalence of depression and anxiety among pregnant women during the COVID-19 pandemic through a systematic search of the literature from December 2019 to February 2021, moderation by time showed that prevalence of anxiety was higher in studies conducted later in the pandemic (18).

Crucially, this increase of anxiety was shown to be directly associated with the pandemic: a study evaluating the basal anxiety (STAI-T) and the state anxiety related to the ongoing pandemic (STAI-S) amoung the same pregnant women in Italy showed that there was a positive association between STAI-T and STAI-S (Pearson = 0.59; *p* ≤ 0.0001) (19). These results suggest that the impact of the pandemic, evaluated at different times in the past months, tended to increase with time.

Additionnaly, a prospective cohort study with 1,367 participants accros USA found an associationbetween high prenatal maternal stress and preterm delivery during COVID-19 pandemic (20). According to the Anglo-Saxon theory of “Prenatal early Life Stress,” there is an increase in Corticotrophin Releasing Hormon (CRH) and cortisol in stressed or depressed mothers (21).

However, cortisol has a deleterious effect on obstetric parameters (prematurity, modification of fetal activity, intrauterine growth retardation) (21). It crosses the placental barrier influencing the development of the fetal nervous system and modifying the programming of the hypothalamic-corticotropic axis of the child which may later be responsible for attention and behavioral disturbances.

After birth, prenatal stress is also associated with disruptions in early dyadic relationships, especially mother-child attachment relationships (22). These direct consequences of prenatal stress during the COVID-19 pandemic are therefore a public health priority, in order to protect maternal mental health and child development.

### Perinatal and Postpartum Depression

This requirement is also valid for perinatal depression, differentiated into prenatal depression (depressive symptoms, for more than 15 days, during pregnancy) and postpartum depression (depressive symptoms, for more than 15 days, in the year following childbirth). These depressive symptoms, defined by DSM5, combine sadness or anhedonia, and four following symptoms including: asthenia, cognitive impairment, negative cognitions, suicidal ideation, psychomotor disturbances, appetite disturbances, and impaired functioning (23). In the general population, outside of a pandemic period, the rate of postpartum depression (PPD) is around 15–20%, and several studies have highlighted an increase in the prevalence of postpartum depression during the pandemic with an estimated prevalence around 22% (24).

A longitudinal study comparing 102 pregnant women and a control group of 102 non-pregnant women showed a significant increase in depression and anxiety in parturients, and an increase in negative affects and a decrease in positive affects greater than in the control group (25). Another study of 1,754 pregnant women in Canada found that women recruited after the onset of the pandemic were almost twice as likely to report symptoms of depression, anxiety disorder, or substance use disorder (26). Finally, a subsequent meta-analysis of 23 studies involving 20,569 participants (16,797 pregnant women and 3,772 postpartum women) during the COVID-19 pandemic shows that 70% of patients present with psychological distress, 31% with depressive symptoms, and 49% with insomnia (24).

The studies suggest that the prevalence of depression appears to be particularly higher in the first and third trimester of pregnancy, with a U-shaped curve (24). The increase in the prevalence rate of depression in the third trimester may be correlated with the proximity of childbirth, and major hormonal changes (27, 28). These initial alarming data were however moderated by the results of a meta-analysis comparing the scores of the Edinburgh Postnatal Depression Survey (EPDS) before and after the pandemic, and finding no significant difference, although the prevalence of postpartum depression tends to be numerically higher during the pandemic (16).

Although these early studies suggest that anxiety-depressive symptoms may have been exacerbated during the COVID-pandemic, results deserve to be considered with caution. The majority of these studies suffer from methodological weaknesses, because of the difficulty in performing screening and prolonged postpartum follow-up. During the perinatal period, patients generally have regular obstetric follow-up, but after childbirth and return home they are mostly isolated, with little medico-social supervision.

Consequently, most of these studies are cross-sectional, and there are few prospective longitudinal studies with prolonged follow-up. In addition, most of the data presented comes from Western countries and China. This characteristic is a limit for their generalization to the whole world. More longitudinal studies, from different countries, are needed to explore these variables during the COVID-19 pandemic.

At any rate, the urgency of appropriate management of these perinatal disorders is justified by their associed morbidity. Perinatal disorders, especially PPD, are associated with a multitude of direct and indirect consequences on mother, infant, siblings, and family. Public Health France and Inserm, two major public health organisms, published on January 6, 2021 the results of the 6th report of the confidential national survey on maternal deaths (ENCMM). It reveals that suicide is the second cause of maternal death (13.4%) for the period 2013–2015 after cardiovascular diseases (13.7%). There is no data regarding the rate of perinatal suicide during the COVID-19 period, but this critical dimension will need to be carefully explored in order to prevent a possible worsening of this dramatic consequence of PPD.

### Acute and Post-traumatic Perinatal Stress Disorder

The notion that childbirth can be traumatic for a third of women and lead to acute stress has been documented in pre-COVID-19 samples. These initial traumatic symptoms are strong predictors of post-childbirth-related post-traumatic stress disorder (CB-PTSD), which is the most chronic manifestation of trauma and has a prevalence of 6–19% (29). This disorder is defined by the presence of traumatic symptoms (nightmares, reliving, avoidance, anxiety) focused on childbirth and pregnancy, and continuing within 1 month after childbirth.

The pandemic has created a more stressful climate in the delivery room during labor and delivery. Factors such as fear of maternal and newborn exposure to the virus during hospital stay, suboptimal preparation for childbirth, feeling of lack of support during childbirth, the limitation of visits to the post-childbirth service and the discrepancy between the expectations in terms of birth before the pandemic and the real experience of childbirth during the pandemic have contributed to a more anxiety-provoking, even traumatic, experience of childbirth (30).

Many studies have evaluated, through web questionnaires mainly, the mother's state of mind the first weeks of post partum to estimate the prevalence of symptoms of PTSD. In one of them, Out of 1,015 pregnant women reached, 737 (72.6%) fully answered the questionnaire and clinically significant PTSD symptoms were present in 75 women (10.2%, NSESSS cutoff 24) (9). Another one showed even higher scores: PTSS rate was 42.9% (IES-R cut-off score ≥ 24). Dismissive and fearful avoidant attachment styles were significantly associated with the risk of depression and PTSS, respectively. Perceived support provided by healthcare staff was a protective factor against depression and PTSS. Another protective factor against PTSS was quiet on the ward due to the absence of hospital visitors (31).

In addition of this increased risk of PTSD after birth, we must take into account the fact that, at the beginning of the pandemic, when little was know about the mother-fetus transmission, some abortions were associated with the infection status of the mothers. In a longitudinal single-arm cohort study conducted in China between May 1 and July 31, 2020 seventy-two pregnant patients with COVID-19 participated in follow-up surveys until 3 months after giving birth (57 cases) or having abortion (15 cases). All cases infected in the first trimester and 1/3 of cases infected in the second trimester had an abortion to terminate the pregnancy, and 22.2% of pregnant patients were suffering from post-traumatic stress disorder or depression at 3 months after delivery or induced abortion (26).

Several characteristics appear to be associated with the risks of developing perinatal mental disorders during a pandemic. First, women from lower socioeconomic categories generally present with more severe anxiety-depressive symptoms, possibly related to increased environmental stressors. Second, multiparous women have a higher prevalence of anxiety and depression than first-time women during the pandemic (24). Pre-existing parenting challenges for multiparous women may play a mediating role here, particularly school closures, increased parenting responsibilities, lost wages, and socioeconomic fragility.

Finally, the presence of previous mental disorders appears to be associated with higher psychiatric symptoms during the pandemic (9). This population of vulnerable women suffering from pre-existing psychiatric disorders is particularly at risk of decompensating their disorder, or of developing psychiatric comorbidities, and as such must be the subject of targeted prevention and support strategies (32).

## Stress Factors Associate With the Fear of the Virus

### Fear of Infection During Pregnancy

The COVID-19 pandemic has generated a wave of fear. The main reaction to this fear has been to avoid places with high risk of contamination, including hospitals. But for pregnant woman, avoiding care facilities was impossible. A dilemma occurred between the need for monthly follow up and the risk of contamination that generated a lot of unanswered questions for mothers:

- “Can I go to antenatal visits?”;
- “Is it better for my fetus that I stay locked up at home?”;
- “Can I keep my plan to deliver the baby in the hospital?.”

Other source of anxiety for pregnant woman was the unknown related to the impact of COVID-19 infection on the fetus, the pregnancy or the mother:

- “Am I more likely to get infected with COVID-19?”;
- “Can the virus be passed to my fetus?”;
- “Am I more likely to develop pregnancy-related complications if I am infected?”;
- “Does Being Pregnant Increase my Risk of Pregnancy Complications?.”

An online study reported that 93% of pregnant women participants reported suffering of an increased source of stress related to COVID-19 infection (33). A number of factors linked to the pandemic may have contributed to the stress during perinatal period, including the perceived vulnerability of parturients to SARS-Cov-2. During perinatal psychiatric consultations, several patients questionned their doctor about the individual risks of contamination. The uncertainty about the potential effects of the virus may have contributed to increase the anxiety of parturients. In previous epidemics such as Severe Acute Respiratory Syndrome (SARS) in 2003, pregnancy may have worsened the clinical course of the infection (34). Current studies point out an increase in ICU admissions and mechanical ventilation for COVID-19 infected pregnant women (35). Moreover, a case study from July 2020 report the presence of coronavirus in both amiotic fluid collected prior to the rupture of menbranes, and in blood drawn early in life (36). A proposed explanation from this study was the lower expression of the angiotensin-converting enzyme 2 receptor and the serine protease TMPRSS2, both necessary for the cell entry and the spread of COVID-19 (37).

Howewer, the first clinical data are reassuring: several newborns that have been tested positive for SARS-CoV-2 or that presented IgM antibodies even when they were delivered by cesarean section or despite an immediate separation from the mother at time of birth didn't have serious complications (38, 39). Moreover, although meta-analysis shows that maternal infection leads to an increased risk of prematurity (19–47% of cases), fetal distress (43% of cases), premature rupture of membranes (19% of cases) and miscarriage (7% of cases), it's hard to know if these complications are produced by the virus itself or caused by the iatrogenic treatment of the infection (40). Finally, the first data in pregnant women rather support a moderate increase in the risk of complications due to COVID-19 infection (41). Taken together, these preliminary studies provide evidence to reassure patients about the direct risks of Sars-Cov-2 infection for pregnancy.

### Vulnerability of Newborn

The second stress factor occurres after childbirth and is related to the vulnerability of the newborn to the unknown virus or about the ways of transmission:

- “Am I at risk of infecting my infant?”;
- “How do I take my baby without risking of infecting him?”
- “Is the virus transmitted through breast milk?”;
- “Can I breastfeed if I have a fever and fear I may have the virus?.”

In the USA, the Centers for Disease Control and Prevention (CDC) initially recommended temporarily separating infected women from their newborns, but the low prevalence of mother-to-newborn transmission lead to new recommendations in December 2020 by the American Academy of Pediatrics (AAP) in support of maintaining the mother and child contact with measures of hand hygiene and wearing of masks.

Regarding the risk of transmission during breastfeeding, first studies were reassuring but did not result in clear recommendations. The American Academic of Pediatrics recommends continuing to promote breastfeeding even with suspected or confirmed COVID-19 (42). Parturients can be reassured about the possibility of breastfeeding their infants, even if they are infected with COVID-19 after birth. Women who develop COVID-19 can be encouraged to breastfeed while applying appropriate respiratory hygiene measures (wearing a mask) and following standard hygiene precautions (disinfecting hands and objects affected).

### Fear of Fear Itself

The third stress factor is related to negatively anticipating consequences of perinatal stress itself for pregnant women. In perinatal clinical practice, women can ask their obstetrician, midwife, or psychiatrist these questions:

- “Will my anxiety disturb my baby's brain?”
- “Will my anxiety interfere with my baby's growth?”
- “Will my child be anxious because I was anxious during my pregnancy?”

This anticipation is associated with a set of biomedical health beliefs in the general population about the impact of the emotions experienced during pregnancy on the development of the fetus, including the theory of the developmental origin of health and disease (DOHaD) (43). This theory suggests that mental (such as emotions or beliefs) and physiological (such as blood sugar or cortisol) states experienced during pregnancy cause similar disorders in children. It assumes that if the mother is anxious, the children will be.

These patients excessively project the consequences of their current anxiety on their baby, imagining for example that their level of stress will cause an anxiety disorder in their child, or will deteriorate its future health. Although there are complex links between mental health during pregnancy and a large number of neurodevelopmental variables, these excessive causal assumptions often play a role in exacerbating anxiety, and cause depressive cognitions such as guilt or shame.

## Stress Factors Associated With *Public Health Policies*

### Social Isolation and Care Restriction

The COVID pandemic has profoundly shaken the daily lives of millions of people: the practices surrounding social rituals such as birth and death were also upset by the the public health policies to minimize the spread of the virus, and have generated social isolation among all the population. The limitation of visits to maternity decided in many countries has caused an increase in social isolation, a reduction in obstetric follow-up, and psychic suffering in many mothers. These situations were associated with several maternal questions:

- “How can I participate to preparation classes with all the restriction?”;
- “Could my partner attend the delivery?”;
- “Can my partner visit me in maternity?”;
- “Will my mother and father be able to see the baby in the maternity?.”

The limitation to access maternity care restrains the preparation for childbirth. Many prenatal interview and preparation sessions for childbirth were canceled or reduced during the pandemic, not allowing a good psychic anticipation (example:).

Also, at the beginning of the pandemic, some maternities decided to deny attendants from going to delivery rooms to support mothers during labor, such as the New York-Presbyterian and Mount Sinai Hospital in the US. These maternities have chosen to minimize family contact with the hospital system to limit the risk of interpersonal transmission, when virus was still new, contamination modes poorly known, and incidence of dramatic infections.

Some maternities have even prohibited visits or the presence of spouses after childbirth and during postpartum hospitalization. Fathers were thus obliged to stay at home during the first days of the child, and mothers found themselves without conjugal support during the immediate postpartum period. This situation was all the more unpleasant as the symptoms of Baby Blues occur during this period of postpartum: the presence of the spouse is important when these transient symptoms occur in order to support mothers in the face of these difficulties. The restriction of postpartum visit have contributed to greater psychological vulnerability and an increased risk of postpartum depression in these patients, as well as consequences for couples (44). Indeed, participating in childbirth and early parenting is associated with immediate and long-term effects for parent and child: this strengthens marital ties, early mother-infant relations, and promotes the harmonious neurodevelopment of the child (45).

In addition, recommended health measures in maternity hospitals (wearing mask, respect social isolation) has sometimes been associated with real obstetric violence: encouragement to wear a mask during expulsion efforts for several women increased the anxiety-provoking climate around childbirth, and could be associated with post-traumatic stress disorder persisting months after the maternity exit (44).

Social support, especially from the spouse, is a major protective factor against postpartum depression (31). For future fathers, meeting their baby several days after birth could also be an important issue. The remoteness of the fathers was able to promote the risks of paternal perinatal depression, designating the prenatal or postpartum depression affecting fathers, in addition to the risk of postpartum depression in mothers (44). In response to the new restriction formulated by several American hospitals on the presence of the spouse during childbirth, a decree of March 28, 2020, following a petition that quickly collected more than 600,000 signatures, finally authorized the presence of a support person in USA.

### Lockdown's Consequences

Second, the generalized lockdown instaured by several countries during the pandemic has been experienced as an extremely uncomfortable experience due to the separation from their loved ones, lack of freedom, and emotional isolation. Studies in the general population have shown a higher prevalence of psychological distress and anxiety-depressive symptoms during lockdown (46, 47).

There were frequently two types of stress factors associated with confinement. The first one is the loss of social resources, coupled with the fear of physical and emotional isolation:

- “What am I going to do if my loved ones cannot come and help me?”;
- “My parents will be able to see the first months of my newborn?”;
- “How can I take care of my oldest child if school is closed?.”

The second one was the loss of physical resources, associated with the anticipation of a food shortage, domestic, or an economic fragility of the household:

- “What will I do if I do not find enough diapers?”;
- “How can I feed this child if I am unemployed?”
- “Will my spouse meet the family's needs?”

The effects of the pandemic on perinatal mental health is also associated with the consequences of confinement among those who have the least social support (48). In an online study in USA, parturients associated their stress primarily with fear of lack of food reserves (59.2%), loss of household income (63.7%), loss of childcare services (56.3%), and conflict between household members (37.5%) (33).

With the closure of school and the isolation of grandparents, many parents have also additional tasks such as babysitting and helping with homeschooling. In this context, single mothers are even more severely affected (22). Single pregnancies, especially for immigrant mothers without immediate family support, were particularly at risk of psychiatric decompensation (44). These mothers who have sometimes already dependent children found themselves isolated at home, without the possibility that the family can come and help them, with all tasksrelated to newborn and siblings.

Violence against women is also an important issue. Although public health guidelines recommend staying at home for safety, the home is the least safe place for these patients. Generalized confinement has been associated with an upsurge in domestic violence, often even appearing in homes where it had not previously taken place. In Canada, calls for violence support services have tripled (49), and these preliminary data are probably underestimated because many victims fear reprisals or are deprived of all contact with the outside world. We can underline the strong entanglement between the decrease in social resources (for both partners), the stress induced by the prospect of loss of economic resources, and domestic violence (49).

**Figure 1:**
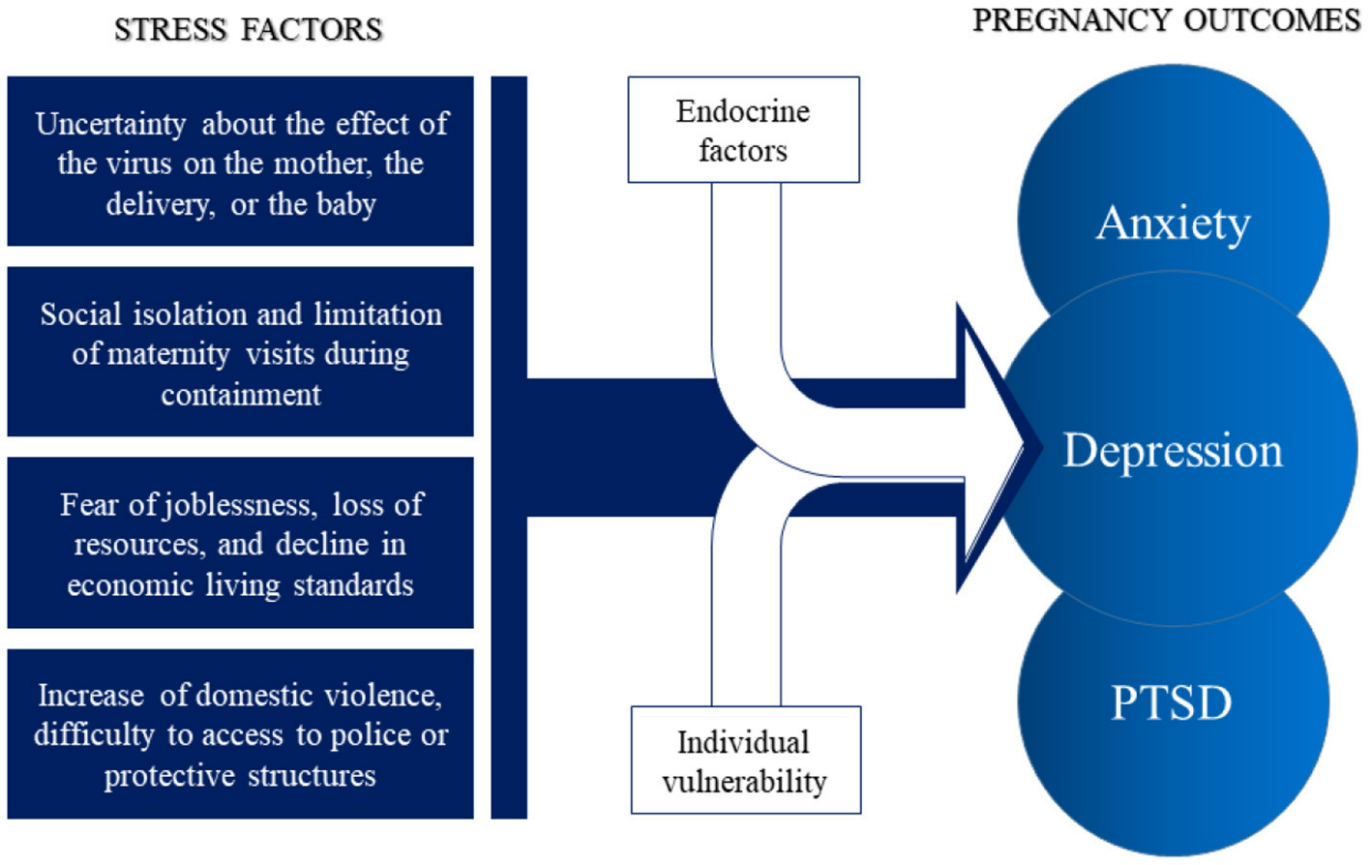
Maternal stress factors and psychiatric outcomes during the COVID-19 pandemic. These stress factors associated with perinatal endocrine factors (variation in cortisol, progesterone, and neurosteroid) and individual vulnerability (domestic violence, social insecurity) may lead to a range of psychiatric symptoms and psychiatric disorder, including anxiety, depression, and post-traumatic stress disorder (PTSD).

On the other side, the lockdown has also caused a redefinition of family life, fundamentally changing its systemic organization. For some, lockdown was an opportunity to forge a new family cohesion based on values perceived as priorities for the household. Because of the lockdown period, fathers had to stay home longer than their paternity leave. This situation has been an opportunity for families to develop strong triadic relationships, to deepen interpersonal relationships, and to develop new partnership skills in the couple. Some couples have also expressed their positive feelings associated with these first postpartum weeks in a period of confinement, with a tightening of the family cocoon around the triad (44) (Figure 1).

### The Vaccination Dilemma

Despite its effectiveness, vaccination no longer enjoys universal support from the population around the world. Indeed, in the Western world, many anti-vaccine groups have emerged, fighting against the recommended vaccines (50, 51). In France, during the 2016 Health Barometer, up to 25% of the population was unfavorable to general vaccination (52). During the pandemic, anti-vaccine positions have increased dramatically, alongside lingering doubt about vaccine safety. These doubts were particularly important for pregnant women fearing effects on their fetus. The following questions were frequently asked related to vaccines:

- “Can I have a miscarriage if I am vaccinated during my pregnancy?”
- “Will my child have congenital malformation if I am vaccinated during my pregnancy?”;
- “Will my child have autisitic spectrum disorder if I am vaccinated during my pregnancy?”;

Since the influenza A (H1N1) pandemic, many debates have taken place on the usefulness, efficacy and safety of a vaccine developed for short-time epidemic (53). This considered short period has been explained by a virus not totally unknown, technologies in development for 30 years, colossal investments and a vaccine tested by a very large panel of population. Many uncertainties appear in connection with the fear of unknown side effects due to the lack of time to carry out long and systematic pharmacovigilance. However, faced with the rapid spread of Covid-19, the paucity of proposed therapeutic strategies and the lack of knowledge obtained on this new virus, a major issue in this pandemic is the rapid development of a safe and effective vaccine. This race against time has generated many doubts about safety and the absence of long-term side effects in the general population. These uncertainties are exacerbated in pregnant women because no data have been available in the beginning of the pandemic, because pregnant women were excluded from clinical studies.

The current data are however reassuring for the vaccination of pregnant women: mRNA vaccines consist of a lipid nanoparticle in which mRNA is encapsulated, allowing it to enter human cells. The vaccine then provides the host cells with the information to make only the S-glycoprotein, a protein that normally allows the virus to attach to human receptors. Our immune system therefore recognizes this S-glycoprotein as a foreign antigen, triggers the immune response and the production of antibodies. MRNA does not enter the cell nucleus, so it cannot modify human DNA and is rapidly degraded in the cell cytoplasm. The first results on animal studies of these vaccines are also reassuring as to the teratogenic risks.

Faced with its data, American College of Obstetrical and Gynecology (ACOG) and the Society for Maternal-Fetal Medicine (SMFM), state that pregnant women who meet criteria for receiving COVID-19 vaccine may be vaccinated (52). The National College of French Gynecologists and Obstetricians (CNGOF) and the French National Authority of Health (HAS) has pleaded for an extension of the anti-COVID-19 vaccination to pregnant women and recommend mRNA vaccines for pregnant women; however the injection during the first trimester should be avoided in principle (54).

## Protect Perinatal Mental Health Face to the Pandemic

### Preventive Specific Support During Pregnancy

The COVID pandemic is forcing us to adapt the organization of perinatal care in order to protect this fragile population against the direct or indirect consequences of the pandemic. It is crucial to prevent the appearance of mental disorders before they can disrupt the behavior of patients: studies have pointed out that anxiety predicted the cancellation of routine obstetrical appointments during lockdown, degrading medical and perinatal follow-up for these women, and restricting the management of their anxiety (55).

We suggest that the implementation of specific perinatal psychiatric care programs during pregnancy is essential to reduce symptoms of anxiety and depression, and prevent the fetal and maternal consequences of these disorders. These strategies should promote close collaboration between midwives, obstetric care, Maternal and Child Health Protection team, and perinatal psychiatric care. This networking between various professionals seems essential to avoid losing sight of suffering women (especially when they are in situations of precariousness, isolation or recent migration), often in difficulty in seeking help from professionals.

Maintaining perinatal follow-up during pregnancy can provide a barrier against the development of pre-partum psychiatric complications (44). The pandemic has drastically reduced access to mental health services, leading to an impoverishment of usual follow-up strategies (56). The early detection of clinical symptoms of anxiety or perinatal depression is a public health objective during this troubled time (17). Overall, the direct and indirect stressors associated with the pandemic that we have discussed are difficult to assess with conventional psychometric tools. The clinical scales are used to screen symptoms of anxiety or depression once they set in, and the disorder has already manifested itself. There is a lack of predictive instruments that can be used during the perinatal period (31).

The PREPS (Pandemic-Related Pregnancy Stress Scale) is a scale that can be used to assess stress factors dimensions related to pandemic: (1) Perinatal Infection Stress, 5 items related to the risk of infection; (2) Preparedness Stress, 7 items related to the stress of preparing for childbirth and postpartum; (3) Positive Appraisal, 3 items related to the favorable experience associated with the pandemic (57). This tool focused on stressors could be systematically used during pre-partum consultations, to early detect situations at risk of developing perinatal psychiatric disorders, before the onset of symptoms (44).

The use of these tools would also make it possible to offer women at risk behavioral strategies for preventing stress, before it becomes overwhelming. Studies pointed out the protective effect of pre-partum physical exercise against anxiety and depressive symptoms (58). Maintaining regular physical activity in the absence of obstetric contraindications is a useful and easy strategy for parturients during the pandemic. Women with proven risk factors should be encouraged to maintain regular physical activity during prenatal period.

### Postpartum Follow-Up and Vulnerable Patients

After childbirth, the need for a specific organization of care is also a challenge during a pandemic. The main problem encountered is the isolation of patients after leaving the maternity ward, increased by lockdown. Continuity of care must be maintained between pediatricians, obstetricians, nurses, midwives and perinatal psychiatry teams, and sometimes in connection with adult psychiatry services. However, the destabilization of the health system during the pandemic weakened this course of common care and perinatal interdisciplinarity.

Most mother-baby psychiatric units in France and Europa had to temporarily suspend their admissions during the crisis, due to uncertainty about the risks of transmission (44). The disorganization of psychiatric care may have been an additional factor in the aggravation of pre-existing clinical pictures, or in the increase in the prevalence of disorders. In the absence of a mother-baby hospitalization unit, severe postpartum depression or puerperal psychosis caused a systematic separation of the mother and the infant: the mother was hospitalized in general psychiatric hospital, and the infant generally kept in nursery. Knowing the serious consequences of perinatal anxiety and postpartum depression on the health of mothers and newborns, responding to this issue during the pandemic presents a real challenge.

During the 2020 lockdown, the Sorbonne University maternities in Paris set up a new care system offering the possibility for women giving birth a 30 min telephone interview with a psychologist at 2 weeks postpartum, and 6 weeks later (59). About 80% of patients benefited from this follow-up during lockdown. The first interview focused on the mother's experience of childbirth and the conditions of discharge at home, associated with screening for perinatal post-traumatic stress disorder (PTSD) using the Perinatal PTSD Questionnaire (PPQ). The second interview more accurately assessed early dyadic relationships [using the New Mother-to-Infant Bonding Scale and Dyadic Adjustment Scale (60)] and symptoms of postpartum depression (using the EPDS scale). These type of protocols have participated in the early detection of risk situations for postpartum depression, allowing targeted intervention during the COVID-19 pandemic (59).

### Digital Platforms in Perinatality

The use of new technologies such as digital platforms (e.g., online phone applications) is an effective way to maintain the contact with patients, and implement early screaning strategies. These tools assess and identify patients with risk factors for psychiatric symptoms, and refer them to teams specializing in perinatal mental health. These digital medical technologies are growing in the medical world, but are still fewly used in perinatal care. This gap was able to be reduced during the pandemic, under the effect of the pressure induced by the lockdown. The need to find perennial tools to assess maternal mental health in postpartum has encouraged an expansion of the use of these digital technologies.

Otherwise, teleconsultation is essential to evaluate the first dyadic interactions and to see the family environment. These home video consultations can be carried out with the dyad, the father, and even the immediate family surrounding the baby, involving the pediatric team and the Child, Maternal and Child Health Protection team if necessary. However, the use of this video technology is only possible in the most developed countries, and in the regions best equipped with digital technology and computer networks. Speaking another language or not having easy access to these technologies can hamper this care. When possible, perinatal team could propose, in conjunction with all early childhood professionals, to set up home visits for the most vulnerable dyads who do not have access to these digital technologies. Regular teleconsultations offered for at-risk patients would be beneficial to ensure this perinatal follow-up.

Morevoer, online application with real-time screening of psychiatric symptoms and regular intervals in the patient's usual environment (home) would be useful. An innovative project currently underway at Sorbonne University proposes an application to strengthen the screening and care of women in the postpartum period. On this platform, patients benefit from practical advice on the primary care (feeding, daily care such as baths and sleep), and medical care (vaccination schedule, and postpartum follow-up interviews).

The application also proposes early, graduated intervention and a connection, if necessary, with a care service. Several levels of therapeutic response are offered on the platform depending on the score of the questionnaires: if no symptoms, regular psycho-educational support; if mild to moderate symptomatology, greater support with psychotherapy exercises (cardiac coherence, cognitive behavioral therapy); if severe symptomatology, contact with a perinatal psychiatry unit. This type of protocol could be systematically offered to patients, and extended on a national level (Table 1).

**Table 1:** Questions frequently asked by mothers during COVID-19 pandemic.

**Mother's questions**	**Caution evidence**	**Reassuring evidence**	**Potential medical advise**
Does Being Pregnant Increase my Risk of Pregnancy Complications?	Some report have found an increased risk of prematurity, fetal distress, premature rupture of membranes and miscarriage for infected mothers	There are no evidence to conclude if pregnancy complications are due to the effect of the virus itself or to the iatrogenic treatment of the infection	The risks associated with the virus for pregnant women are limited when patients have regular perinatal follow-up
Can the virus be passed from me to my fetus?	The first studies are reassuring with a low rate of transplacental passage, but this question requires further evidence to confirm transmission hypothesis	When cases of suspected transmission during pregnancy have been reported, newborn tested positive to COVID-19 didn't had serious complication	The risk associated with transplacental transmission or contamination at birth is low, and mothers can be reassured about the risks to their infants
Can I breastfeed if I have fever and fear I have the virus?	There are still few studies on the transmission of the virus from mother to child during breastfeeding, and more scientific work should be done on this topic	The American Academic of Pediatrics is very reassuring and recommends breastfeeding even if infected by COVID-19	During breastfeeding, women have to applying appropriate respiratory hygiene measures (wearing a mask) and following standard hygiene precautions (disinfecting the hands and objects affected)
Can I get vaccinated during my pregnancy?	Pregnant individuals were excluding from the clinical trials for Pfizer and Moderna vaccines but results of animal studies on these two vaccines are reassuring	The American college of Obstetricians and Gynecologists state that pregnant women can be vaccinated	Mothers should give priority to vaccination in front of the risk-benefit balance in favor of its implementation, and can be reassured about the associated thromboembolic risks
What are my options to cope with my perinatal stress during the pandemic?	Studies have shown an increase of anxiety and depression during the pandemic, and its symptoms have been experienced by many women during the perinatal period. It's more difficult to deal with these symptoms in countries with precarious health systems	In many country, new tools have been developed to protect and support mothers in the face of stressors linked to the pandemic, such as regular teleconsultations and the use of online applications after maternity discharge	Mothers can be encouraged to contact perinatal psychiatric services when these exist in the structures where they are taken care of. When they are not available, a quick contact with a psychiatrist or the general practitioner should be organized in order to intervene early on the emerging symptoms.

*We describe here questions frequently asked by pregnant women or mothers who have just given birth, and summarize the data currently available in the scientific literature, and medical advice that can be given in response. Its recommendations are based on the current state of the literature, and are likely to change over time*.

## Conclusion

The COVID-19 pandemic causes harm consequences for perinatal mental health, and special attention should be paid to parturients and mothers during this time. Pregnant and postpartum women are particularly at risk of developing psychiatric symptoms during the pandemic. Social support appears to be a major protective factor against these disorders, and could be promoted by the use of new digital technologies, video teleconsultations, andconnected applications. Targeted prevention strategies should be systematically offered to women in order to detect clinical symptoms early, and to offer rapid therapeutic interventions.

## Author Contributions

HB and LJ: conceptualization, methodology, supervision, project administration, and funding acquisition. HB, BV, CJ, and LJ: resources. HB: writing—original draft preparation. HB, BV, CJ, RT, and LJ: writing—review and editing. All authors have read and agreed to the published version of the manuscript.

## Conflict of Interest

The authors declare that the research was conducted in the absence of any commercial or financial relationships that could be construed as a potential conflict of interest.

## Publisher's Note

All claims expressed in this article are solely those of the authors and do not necessarily represent those of their affiliated organizations, or those of the publisher, the editors and the reviewers. Any product that may be evaluated in this article, or claim that may be made by its manufacturer, is not guaranteed or endorsed by the publisher.
